# Hepatic Crown-Like Structure: A Unique Histological Feature in Non-Alcoholic Steatohepatitis in Mice and Humans

**DOI:** 10.1371/journal.pone.0082163

**Published:** 2013-12-11

**Authors:** Michiko Itoh, Hideaki Kato, Takayoshi Suganami, Kuniha Konuma, Yoshio Marumoto, Shuji Terai, Hiroshi Sakugawa, Sayaka Kanai, Miho Hamaguchi, Takahiro Fukaishi, Seiichiro Aoe, Kazunari Akiyoshi, Yoshihiro Komohara, Motohiro Takeya, Isao Sakaida, Yoshihiro Ogawa

**Affiliations:** 1 Department of Organ Network and Metabolism, Tokyo Medical and Dental University, Tokyo, Japan; 2 Department of Molecular Endocrinology and Metabolism, Graduate School of Medical and Dental Sciences, Tokyo Medical and Dental University, Tokyo, Japan; 3 Japan Science and Technology Agency, PRESTO, Tokyo, Japan; 4 Medical Research Laboratories, Shionogi & Co. Ltd., Osaka, Japan; 5 Department of Gastroenterology and Hepatology, Yamaguchi University Graduate School of Medicine, Yamaguchi, Japan; 6 Heart Life Hospital, Okinawa, Japan; 7 Department of Home Economics, Otsuma Women's University, Tokyo, Japan; 8 Department of Polymer Chemistry, Kyoto University Graduate School of Engineering, Kyoto, Japan; 9 Department of Cell Pathology, Graduate School of Medical Sciences, Kumamoto University, Kumamoto, Japan; Institute of Medical Research A Lanari-IDIM, University of Buenos Aires-National Council of Scientific and Technological Research (CONICET), Argentina

## Abstract

Although macrophages are thought to be crucial for the pathogenesis of chronic inflammatory diseases, how they are involved in disease progression from simple steatosis to non-alcoholic steatohepatitis (NASH) is poorly understood. Here we report the unique histological structure termed “hepatic crown-like structures (hCLS)” in the mouse model of human NASH; melanocortin-4 receptor deficient mice fed a Western diet. In hCLS, CD11c-positive macrophages aggregate to surround hepatocytes with large lipid droplets, which is similar to those described in obese adipose tissue. Histological analysis revealed that hCLS is closely associated with activated fibroblasts and collagen deposition. When treatment with clodronate liposomes effectively depletes macrophages scattered in the liver, with those in hCLS intact, hepatic expression of inflammatory and fibrogenic genes is unaffected, suggesting that hCLS is an important source of inflammation and fibrosis during the progression of NASH. Notably, the number of hCLS is positively correlated with the extent of liver fibrosis. We also observed increased number of hCLS in the liver of non-alcoholic fatty liver disease/NASH patients. Collectively, our data provide evidence that hCLS is involved in the development of hepatic inflammation and fibrosis, thereby suggesting its pathophysiologic role in disease progression from simple steatosis to NASH.

## Introduction

Non-alcoholic fatty liver disease (NAFLD) is one of the most common forms of chronic liver disease closely related to the metabolic syndrome and type 2 diabetes mellitus [1], [2]. The clinical spectrum of NAFLD ranges from simple hepatic steatosis to non-alcoholic steatohepatitis (NASH), the latter of which can progress to cirrhosis and hepatocellular carcinoma [3]. According to the “two-hit” hypothesis, the pathogenesis of NASH may involve at least two processes; excessive accumulation of lipids in the liver as the 1^st^ hit plus additional pathogenic stimuli as the 2^nd^ hit, such as proinflammatory cytokines, oxidative stress, endotoxins, and lipotoxicity [4], [5], [6], [7]. Hepatic macrophages are a major source of proinflammatory mediators such as tumor necrosis factor-α (TNFα), interleukin-6, and reactive oxygen species, which are considered to accelerate hepatic steatosis and insulin resistance [8], [9]. There is evidence that macrophages are involved in the pathogenesis of some rodent models of experimentally-induced liver fibrosis [10], [11]. However, the pathophysiologic role of macrophages in the development of NASH is still unclear; it is partly because the limited availability of suitable animal models that reflect a liver condition of human NASH [12]. For instance, chemically-induced liver fibrosis is not accompanied by obesity, insulin resistance, and hepatic steatosis [12]. Dietary deficiency of methionine and choline also develops steatosis and mild fibrosis, without obesity and insulin resistance [12].

We have recently reported that melanocortin-4 receptor (MC4R) deficient (MC4R-KO) mice on a high-fat diet (HFD) exhibit a liver condition similar to human NASH, which is associated with obesity, insulin resistance, and dyslipidemia [13]. They also develop well-differentiated hepatocellular carcinoma after a longer period of time [13]. Moreover, they show enhanced adipose tissue inflammation characterized by macrophage infiltration and adipocytokine dysregulation [13], which may contribute to excessive lipid accumulation and enhanced fibrosis in the liver [14], [15], [16], [17], [18]. MC4R is a seven-transmembrane G protein-coupled receptor that is expressed in the hypothalamic nuclei implicated in the regulation of food intake and body weight [19]. Because MC4R mRNA expression is restricted to the hypothalamus and other brain regions and is undetectable in the liver and the adipose tissue [20], it is likely that the hepatic phenotype in MC4R-KO mice results from loss of function of MC4R in the brain rather than in the liver itself. In line with this, Nogueiras *et al.* reported that MC4R signaling in the brain may regulate lipid metabolism in the liver [21]. Collectively, MC4R-KO mice would provide a novel mouse model of NASH with which to investigate the sequence of events that comprise diet-induced steatosis and fibrosis in the liver.

Evidence has accumulated indicating that obesity is a state of chronic, low-grade inflammation, which may play a role in the pathogenesis of obesity-related complications [22]. There is also considerable evidence that macrophages are infiltrated into obese adipose tissue to induce inflammatory responses [22], [23], [24]. Recent studies have pointed to the heterogeneity of macrophages in obesity; *i.e.* M1 or “classically activated” (proinflammatory) macrophages and M2 or “alternatively activated” (anti-inflammatory) macrophages [25], [26]. Adipose tissue macrophages in lean mice are polarized toward the M2 activation state, whereas in obese adipose tissue, they are toward the M1 activation state [25]. Histologically, M2 macrophages are scattered in the interstitial spaces between adipocytes [26]. On the other hand, CD11c-positive M1 macrophages aggregate to constitute crown-like structures (CLS) in obese adipose tissue of humans and rodents, where they are considered to scavenge the residual lipid droplets of dead adipocytes [27]. Notably, the number of CLS is positively correlated with systemic hyperinsulinemia and insulin resistance in obese subjects [28], [29], suggesting the pathophysiologic role of CLS in adipose tissue inflammation.

Here we report that CD11c-positive macrophages aggregate to constitute CLS-like structures surrounding hepatocytes with large lipid droplets in the liver from MC4R-KO mice fed a Western diet (WD), which may be referred to as “hepatic CLS (hCLS)”. Notably, the number of hCLS is positively correlated with the extent of liver fibrosis in our NASH model. We also observed increased number of hCLS in the liver of NAFLD/NASH patients, whereas it is rarely detected in patients with chronic viral hepatatis. Collectively, our data provide evidence that hCLS is critically involved in the development of hepatic inflammation and fibrosis, thereby suggesting its pathophysiologic role in disease progression from simple steatosis to NASH.

## Materials and Methods

### Ethics Statement

All animal experiments were conducted in accordance to the guidelines of Tokyo Medical and Dental University Committee on Animal Research (No. 2011-207C, No. 0130269A). The clinical study protocol was approved by the ethical committee on human research of Tokyo Medical and Dental University, Yamaguchi University Hospital, and Heart Life Hospital (No. 1366 and No. 1397, Medical Research Ethics Committee of Tokyo Medical and Dental University; H24–80, Institutional Review Board of Yamaguchi University Hospital; 24-6, Institutional Review Board of Heart Life Hospital). The study is a follow-back study using only existing materials and information including human biological specimens. All samples were collected and stored for clinical practice at Yamaguchi University Hospital and Heart Life Hospital. Although written informed consent was not obtained for the current study, we obtained approval from Ethics Committee/Institutional Review Board of each institution based on Japanese Ethical Guidelines for Clinical Studies, disclosed the detailed information on the study protocol, and provided all participants with an opportunity to refuse their inclusion in the study.

### Animals

The MC4R-KO mice on the C57BL/6J background were a generous gift from Dr. Joel K. Elmquist (University of Texas Southwestern Medical Center) [19]. Male C57BL/6J wildtype mice were purchased from CLEA Japan (Tokyo, Japan). The animals were acclimated to the environment in a temperature-, humidity-, and light-controlled room (12-h light and 12-h dark cycle) and allowed free access to water and a standard chow (CE-2; 343.1 kcal/100 g, 12.6% energy as fat; CLEA Japan) for one week. Eight week-old male mice were fed a WD (D12079B; 468 kcal/100 g, 41% energy as fat, 34.0% sucrose, 0.21% cholesterol; Research Diets, New Brunswick, NJ) or a HFD (D12492; 524 kcal/100 g, 60% energy as fat, 8.9% sucrose, 0.03% cholesterol; Research Diets). Methionine and choline-deficient diet (#518810; Dyets, Bethlehem, PA) was used to induce steatohepatitis. At the end of the experiment, they were sacrificed, when fed *ad libitum*, under intraperitoneal pentobarbital anesthesia (30 mg/kg).

### Blood Analysis

Blood glucose concentrations and serum concentrations of alanine aminotransferase (ALT), total cholesterol (TC), triglyceride (TG), free fatty acid, and insulin were measured as previously described [13].

### Hepatic TC and TG Content

Total lipids in the liver were extracted with ice-cold 2∶1 (vol/vol) chloroform/methanol. The TC and TG concentrations were measured by an enzymatic assay kit (Wako Pure Chemicals, Osaka, Japan) [13].

### Histological Analysis

The liver was fixed with neutral-buffered formalin and embedded in paraffin. Four-µm-thick sections of the liver were stained with Masson-trichrome and Sirius red [13]. Immunohistochemical staining for F4/80, α-smooth muscle actin (αSMA) (ab5694, Abcam, Cambridge, UK), and type I collagen (1310-01, Southern Biotech, Birmingham, AL) were performed [30]. Positive areas for Sirius red, αSMA, and F4/80 were measured using the software WinROOF (Mitani, Chiba, Japan) [13]. The number of hCLS was counted in the whole area of each F4/80-stained section and expressed as the mean number/mm^2^. For immunofluorescent staining, the liver was embedded in OCT compound and frozen in dry ice-acetone. Ten-µm-thick frozen sections were stained with antibodies against F4/80, type I collagen, fibroblast specific protein 1 (FSP1) (ab27957, Abcam), glial fibrillary acidic protein (GFAP) (Z0334, Dako, Glostrup, Denmark), CD11c (14-0114, eBioscience, San Diego, CA), and secondary antibodies conjugated with AlexaFluor 488, 568 or 594 (Invitrogen, Carlsbad, CA) and Alexa Fluor 647 (Jackson ImmunoResearch Labolatories, West Grove, PA). Lipid droplets were defined by BODIPY 493/503 (Invitrogen). Sections were mounted in Vectashield mounting medium with DAPI (VectorLabs, Burlingame, CA) and photographed using confocal laser-scanning microscope FV10i-DOC (Olympus, Tokyo, Japan).

### Electron Microscopy

Liver samples were fixed in 2.5% glutaralehyde in 0.1 M cacodylate buffer for 1 hour, and postfixed in 1% osmium tetroxide. After dehydration in a graded series of ethanol solutions and propylene oxide and embedding in Epon 812, ultrathin sections were cut by use of an Ultratome, stained with uranyl acetate and lead citrate, and observed with a Hitachi H-7500 electron microscope (Hitachi, Tokyo, Japan).

### Macrophage depletion experiment with clodronate liposomes

Clodronate liposomes were prepared as described previously [31]. In brief, phosphatidylcholine from egg (Avanti Polar Lipids, Alabaster, AL) and cholesterol (Wako Pure Chemicals, Osaka, Japan) were dissolved in chloroform in a glass tube, followed by evaporation of chloroform using nitrogen gas, resulting in a thin layer film. The tube containing the film was dried in a desiccator overnight. Clodronate disodium (Sigma, St. Louis, MO) dissolved in phosphate-buffered saline (PBS) was added to the tube containing the film, and then liposomes were generated by vortexing. The liposome-containing solution was frozen and thawed three times, and subsequently passed through an extruder (Avanti) with a 400 nm membrane. After centrifugation at 10,000× g for 15 minutes, clodronate liposomes were suspended in sterilized PBS. To deplete macrophages, MC4R-KO and wildtype mice fed a WD for 4 or 20 weeks received 0.1 ml of clodronate or PBS liposomes via the tail vein at 6 and 2 days before the end of the experiment.

### Quantitative Real-Time PCR

Total RNA was extracted from the liver using Sepasol reagent (Nacalai Tesque, Kyoto, Japan). Quantitative real-time PCR was performed with StepOnePlus Real-time PCR System using Fast SYBR Green Master Mix Reagent (Applied Biosystems, Foster City, CA) as described previously [32]. Primers used in this study were described elsewhere [13].

### Human Study

Fifty-one Japanese NAFLD patients who had sustained liver dysfunction and 15 chronic viral hepatitis patients were recruited at Yamaguchi University hospital and Heart Life Hospital. We measured body mass index (BMI), and determined plasma concentrations of aspartate aminotransferase (AST) and ALT according to the standard procedures. Liver samples were obtained by ultrasound-guided liver biopsy to evaluate liver histology [33]. Formalin-fixed and paraffin embedded liver specimens were stained with anti-CD68 antibody (M0876, Dako). The liver histology was assessed by two investigators without knowledge of the origin of the slides according to the NASH clinical research network scoring system, including the scores for hepatic steatosis, lobular inflammation, ballooning degeneration and the fibrosis stage [34]. The number of hCLS was counted in the whole area of each CD68-stained section and expressed as the mean number/mm^2^.

### Statistical Analysis

Data are presented as mean ± SE, and *P*<0.05 was considered statistically significant. Differences between two groups were compared using Student *t*-test. Pearson correlation coefficient was employed to investigate the correlation among hCLS number, F4/80-positive area, and fibrosis area. Tests for linear trend were calculated by assigning the average numbers of hCLS for each score of hepatic steatosis, lobular inflammation, ballooning degeneration, and fibrosis stage treated as a continuous variable by using linear regression models. All data were analyzed with Stat View version 5.0 or JMP version 10.0 (SAS Institute Inc, Cary, NC).

## Results

### Hepatic phenotypes of MC4R-KO mice

**Figure 1:**
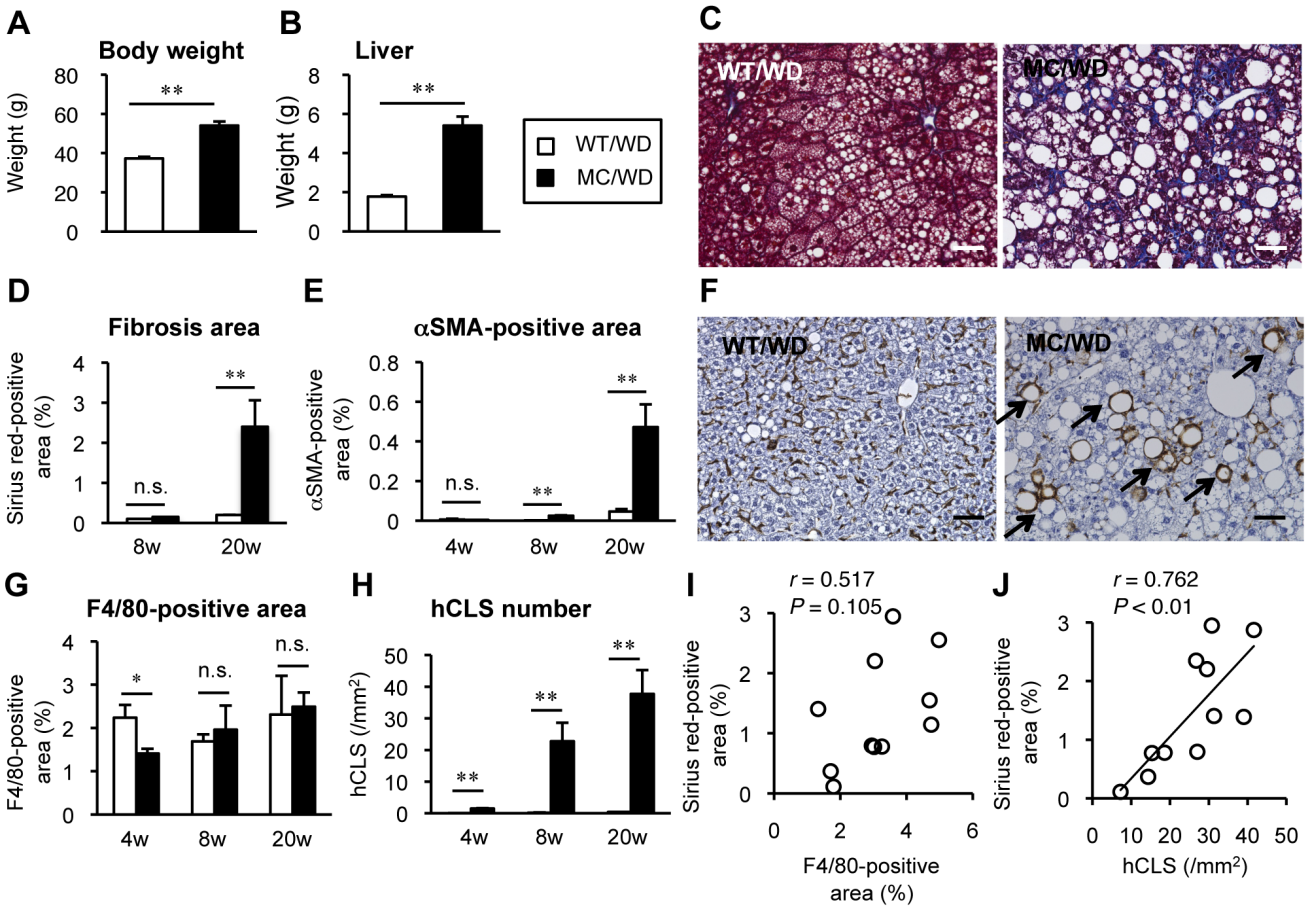
hCLS formation by macrophages and liver fibrosis in MC4R-KO mice fed a WD. Body weight (A) and liver weight (B) of male MC4R-KO (MC) and wildtype (WT) mice fed a western diet (WD) for 20 weeks. (C) Masson-trichrome staining of the liver sections from MC4R-KO and wildtype mice after 20 weeks of WD feeding. Time-dependent changes in liver fibrosis (Sirius red-positive area) (D) and activated fibroblasts (αSMA-positive area) (E) during WD feeding. (F) F4/80 staining at 20 weeks. Characteristic histological features by macrophage, hepatic crown-like structures (hCLS), in the liver from MC4R-KO mice were indicated by arrows. Time-dependent changes in F4/80-positive area (G) and hCLS number (H) during WD feeding. Correlation of fibrosis area with F4/80-positive area (I) and hCLS number (J). Scale bars, 50 µm. * *P*<0.05, ** *P*<0.01, n.s., not significant. *n* = 5–7.

The MC4R-KO mice showed marked increase in body weight and liver weight relative to wildtype mice after 20-week WD feeding (Figure 1A and 1B). They also exhibited increased concentrations of insulin, TC, and ALT and hepatic accumulation of TC as well as TG (Table 1). At this time point, microvesicular steatosis was observed uniformly in the liver from wildtype mice, where inflammatory cell infiltration and tissue fibrosis were rarely observed (Figure 1C). On the other hand, livers from MC4R-KO mice fed a WD exhibited micro- and macrovesicular steatosis, ballooning degeneration, massive infiltration of inflammatory cells, and marked pericellular fibrosis (Figure 1C) as we previously reported using a HFD [13]. Although the area of liver fibrosis was not increased in wildtype mice throughout the experimental period, MC4R-KO mice developed obvious liver fibrosis at 20 weeks of WD feeding (*P*<0.01, Figure 1D). Moreover, the area of αSMA-positive activated fibroblasts was significantly increased in MC4R-KO mice relative to wildtype mice at 8 and 20 weeks (Figure 1E) [13]. We next examined the distribution of macrophages in the liver with F4/80 immunostaining, a representative macrophage marker. In wildtype mice fed a WD, macrophages showed scattered distribution in the liver (Figure 1F). On the other hand, in MC4R-KO mice fed a WD, macrophages aggregated to surround hepatocytes with large lipid droplets (Figure 1F). Given the structural similarity to CLS in obese adipose tissue, it may be referred to as “hepatic CLS”. Although the area positive for F4/80 immunostaining was roughly comparable between the genotypes throughout the experimental period (Figure 1G), the number of hCLS was significantly increased in MC4R-KO mice relative to wildtype mice at 4 weeks, when liver fibrosis was not evident, and thereafter increased time-dependently up to 20 weeks (Figure 1H). Notably, after 20-week WD, the number of hCLS, not the F4/80-positive area, was positively correlated with the extent of liver fibrosis (Figure 1I and 1J).

**Table 1:** Serological parameters and hepatic lipid content of MC4R-KO and WT mice fed a WD for 20 weeks.

	WT		MC4R-KO	
	SD	WD	SD	WD
BG (*ad lib*, mg/dL)	147.7±0.8	195.3±7.2^*^	156.6±16.8	151.4±7.1^§^
Insulin (*ad lib*, ng/mL)	0.47±0.15	1.10±0.11	3.41±1.16	4.72±1.84^*^
TC (mg/dL)	93.7±4.4	204.3±8.7^*^	130.2±11.2	352.1±35.1^*§†^
TG (mg/dL)	112.5±18.2	48.9±3.7^*^	146.3±25.5	69.9±10.1^†^
FFA (mEq/L)	0.38±0.04	0.32±0.01	0.61±0.06^*^	0.52±0.03^*§^
ALT (IU/L)	30.0±1.4	43.4±10.9	56.8±18.7	268.7±34.8^*§†^
Liver TC (mg/g tissue)	1.88±0.46	1.64±0.24	2.58±0.46	4.83±1.14^*§^
Liver TG (mg/g tissue)	37.9±8.3	54.5±6.9	61.0±10.4	133.6±35.8^*^

± SE. ^*^ *P*<0.05 vs. WT-SD; ^§^ *P*<0.05 vs. WT-WD; ^†^ *P*<0.05 vs. MC4R-SD. *n* = 5-7. WT, wildtype; SD, standard diet; WD, western diet; BG, blood glucose; TC, total cholesterol; TG, triglyceride; FFA, free fatty acid; ALT, alanine aminotransferase. Data are expressed as the mean

We confirmed the results in MC4R-KO mice fed a HFD for 20 weeks (Figure S1A-S1C). hCLS was also observed in the liver from wildtype mice fed a HFD for one year, at which they develop liver fibrosis comparable to MC4R-KO mice on a HFD for 20 weeks (Figure S1D and S1E). Furthermore, hCLS was also present in mice fed a methionine and choline-deficient diet, a well-known model of steatohepatitis (Figure S1F and S1G). Collectively, these observations suggest that hCLS is a common histological feature in steatohepatitis models, which precedes the development of collagen deposition and reflects the extent of liver fibrosis.

### Histological characterization of hCLS in MC4R-KO mice

**Figure 2:**
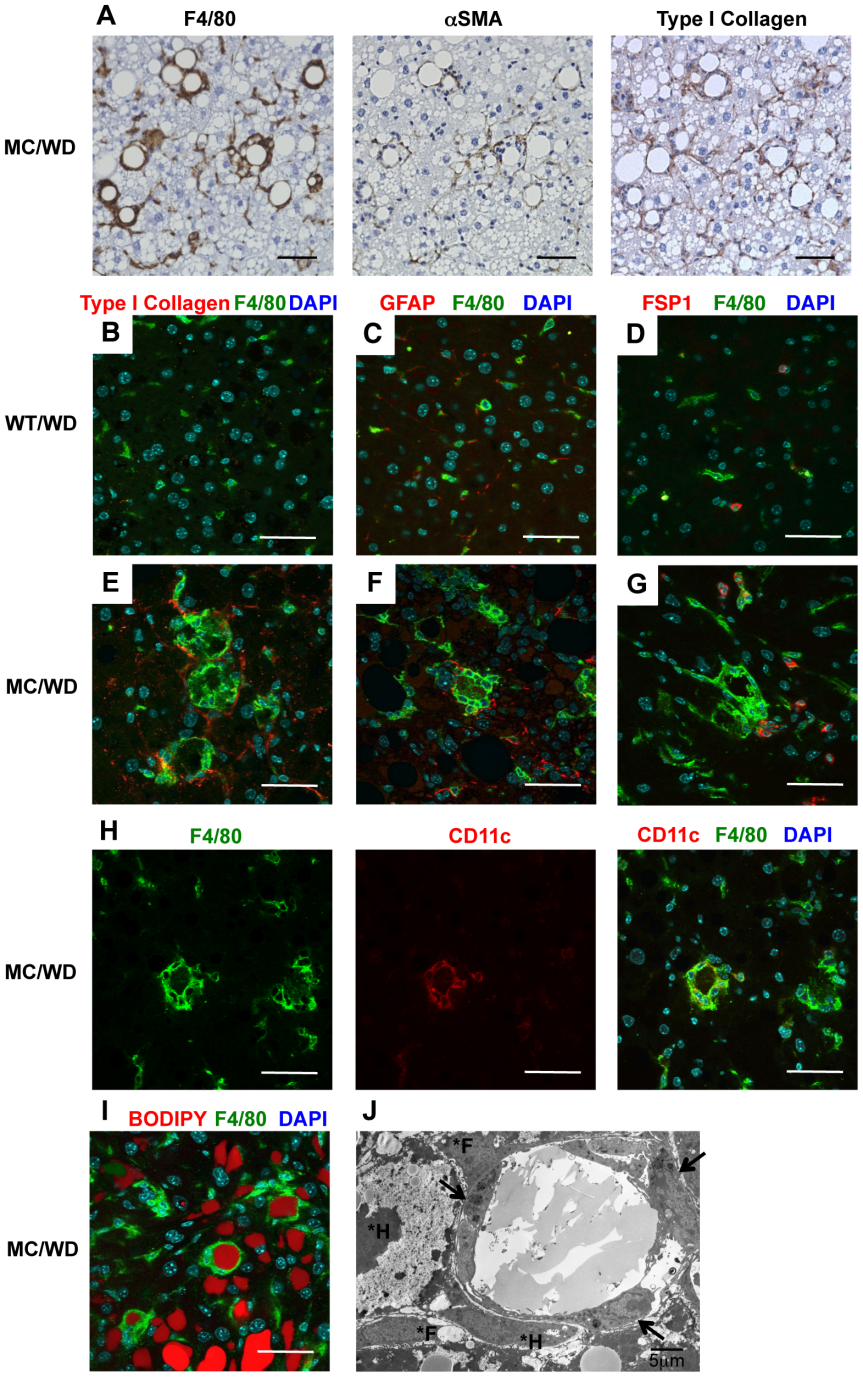
Histological analysis of hCLS in the liver from MC4R-KO mice fed a WD. (A) Serial sections of the liver from MC4R-KO mice fed a WD for 20 weeks stained with F4/80, αSMA, and type I collagen antibodies. Immunofluorescent analysis for F4/80 (B-G), type I collagen (B, E), glial fibrillary acidic protein (GFAP) (C, F), and fibroblast specific protein 1 (FSP1) (D, G) in the liver from wildtype and MC4R-KO mice at 20 weeks. (H) Immunofluorescent analysis for F4/80 and CD11c. (I) Immunofluorescent analysis for F4/80 and lipid droplet (BODIPY). The nuclei were counterstained with DAPI (B–I). Scale bars, 50 µm. (J) Electron micrograph of hCLS. Aggregated macrophages around a lipid droplet (arrows). Fibroblasts (*F) and hepatocytes (*H) are detected around hCLS.

To investigate the histological characteristics of hCLS, we performed immunohistochemical analysis using the liver from wildtype and MC4R-KO mice fed a WD for 20 weeks. The serial liver sections stained with antibodies against F4/80, αSMA, and type I collagen revealed that αSMA-positive myofibroblasts and collagen deposition are located in proximity to hCLS in the liver from MC4R-KO mice (Figure 2A). There were no apparent collagen deposition in the liver from wildtype mice (data not shown). By double-immunofluorescent staining of F4/80 (green) and type I collagen (red), we also observed the adjacent spatial relationship between hCLS and fibrogenic lesions (Figure 2B and 2E). Since previous reports pointed to the heterogeneity of fibrogenic cells during the development of liver fibrosis [35], [36], [37], we examined the expression of GFAP and FSP1, markers for hepatic stellate cells and fibroblasts, respectively. The GFAP-positive cells were diffusely located along the sinusoids in wildtype and MC4R-KO mice (Figure 2C and 2F). The FSP1-positive cells accumulated around hCLS in MC4R-KO mice, whereas they were only sparsely observed in wildtype mice (Figure 2D and 2G). Notably, CD11c was positive only in macrophages that constitute hCLS (Figure 2H). In this study, BODIPY staining revealed that hepatocytes surrounded by hCLS have large lipid droplets (Figure 2I). In electron microscopic analysis, some macrophages having cell processes on the surface and lysosomes in the cytoplasm, assembled around lipid droplets to form hCLS (Figure 2J). Taken together, our data suggest that hCLS is associated with fibrogenic lesions in the liver from MC4R-KO mice on a WD.

### Effect of clodronate liposomes on inflammation and fibrosis in MC4R-KO mice

**Figure 3:**
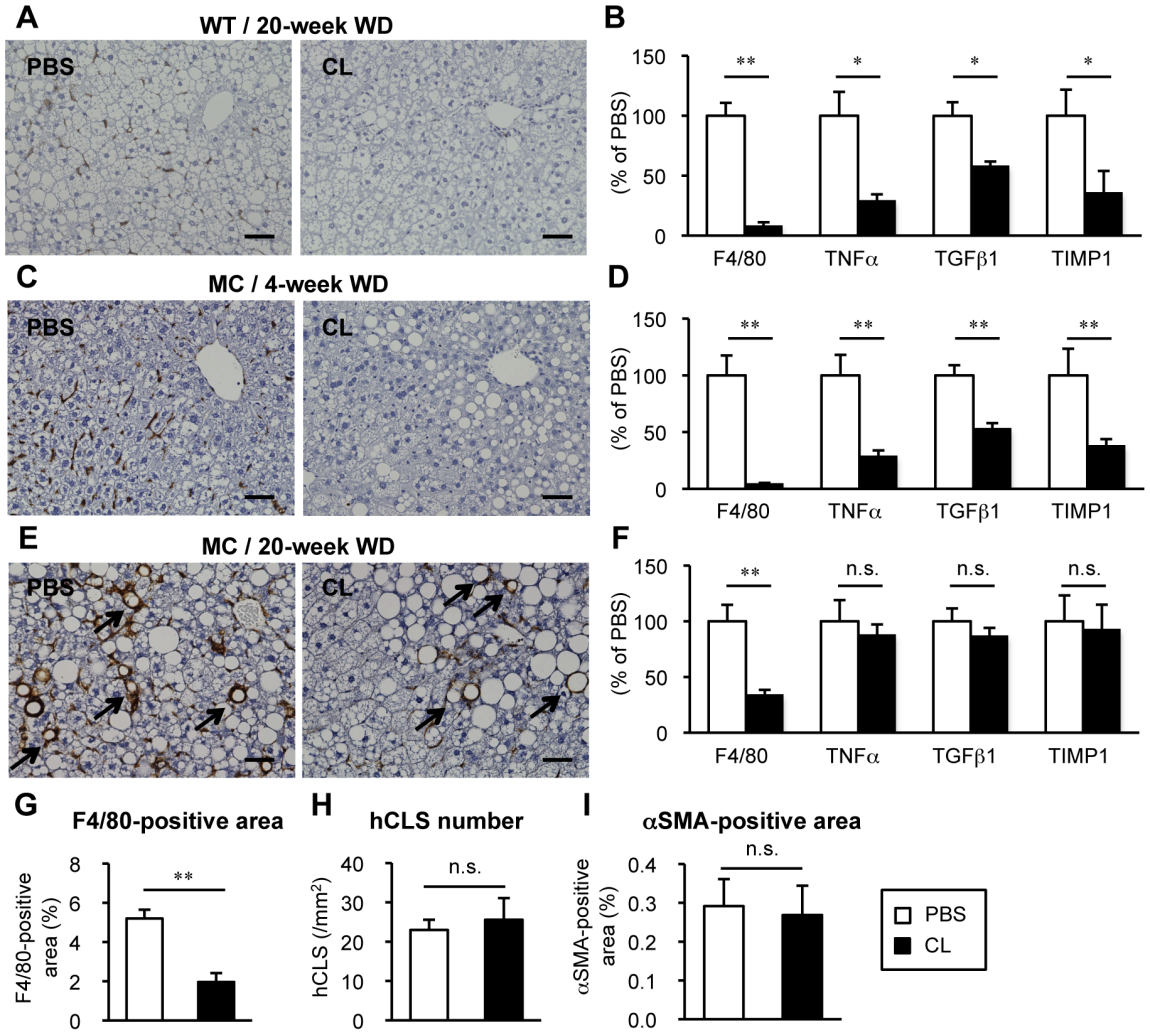
Effect of Macrophage depletion on inflammatory and fibrotic changes in the liver during WD feeding. Representative F4/80 immunostaining (A) and hepatic mRNA expression of inflammatory markers (F4/80, and tumor necrosis factor α (TNFα) and fibrogenic factors (transforming growth factor-β1 (TGFβ1) and tissue inhibitor of metalloproteinase-1 (TIMP1)) (B) in the liver from wildtype mice fed a WD for 20 weeks, at which wildtype mice showed simple steatosis. Representative F4/80 immunostaining (C, E) and hepatic mRNA expression levels (D, F) in the liver from MC4R-KO mice fed a WD for 4 (C, D) and 20 weeks (E, F), at which MC4R-KO mice showed simple steatosis and NASH respectively. Arrows indicate hCLS. Quantification of F4/80-positive area (G), hCLS number (H) and αSMA-positive area (I) at 20 weeks. PBS, PBS liposome; CL, clodronate liposome. Scale bars, 50 µm. * *P*<0.05, ** *P*<0.01, n.s., not significant. *n* = 5–7.

We next examined the functional role of hCLS in hepatic inflammation and fibrosis in MC4R-KO mice on a WD. Administration of clodronate liposomes effectively depleted F4/80-positive macrophages in the steatotic liver from wildtype mice fed a WD for 20 weeks (Figure 3A). Expression of mRNAs for inflammatory genes (F4/80 and TNFα) and fibrogenic genes (transforming growth factor-β1 and tissue inhibitor of metalloproteinase-1) was significantly reduced by treatment with clodronate liposomes (Figure 3B). We also confirmed similar results in the liver from MC4R-KO mice fed a WD for 4 weeks, when they showed simple hepatic steatosis (Figure 3C and 3D). In MC4R-KO mice fed a WD for 20 weeks, macrophages showing scattered distribution in the liver were also depleted by the treatment (Figure 3E). However, macrophages constituting hCLS were resistant to the treatment with clodronate liposomes (Figure 3E). In this setting, treatment with clodronate liposomes resulted in no significant changes in mRNA expression of TNFα and fibrogenic genes (Figure 3F). We confirmed that F4/80-positive area was significantly decreased by the treatment with clodronate liposomes, whereas the number of hCLS and the αSMA-positive area were unchanged (Figure 3G–3I). These observations suggest that hCLS is an important source of hepatic inflammatory and fibrogenic mediators in MC4R-KO mice on a WD.

### hCLS in human NASH

**Figure 4:**
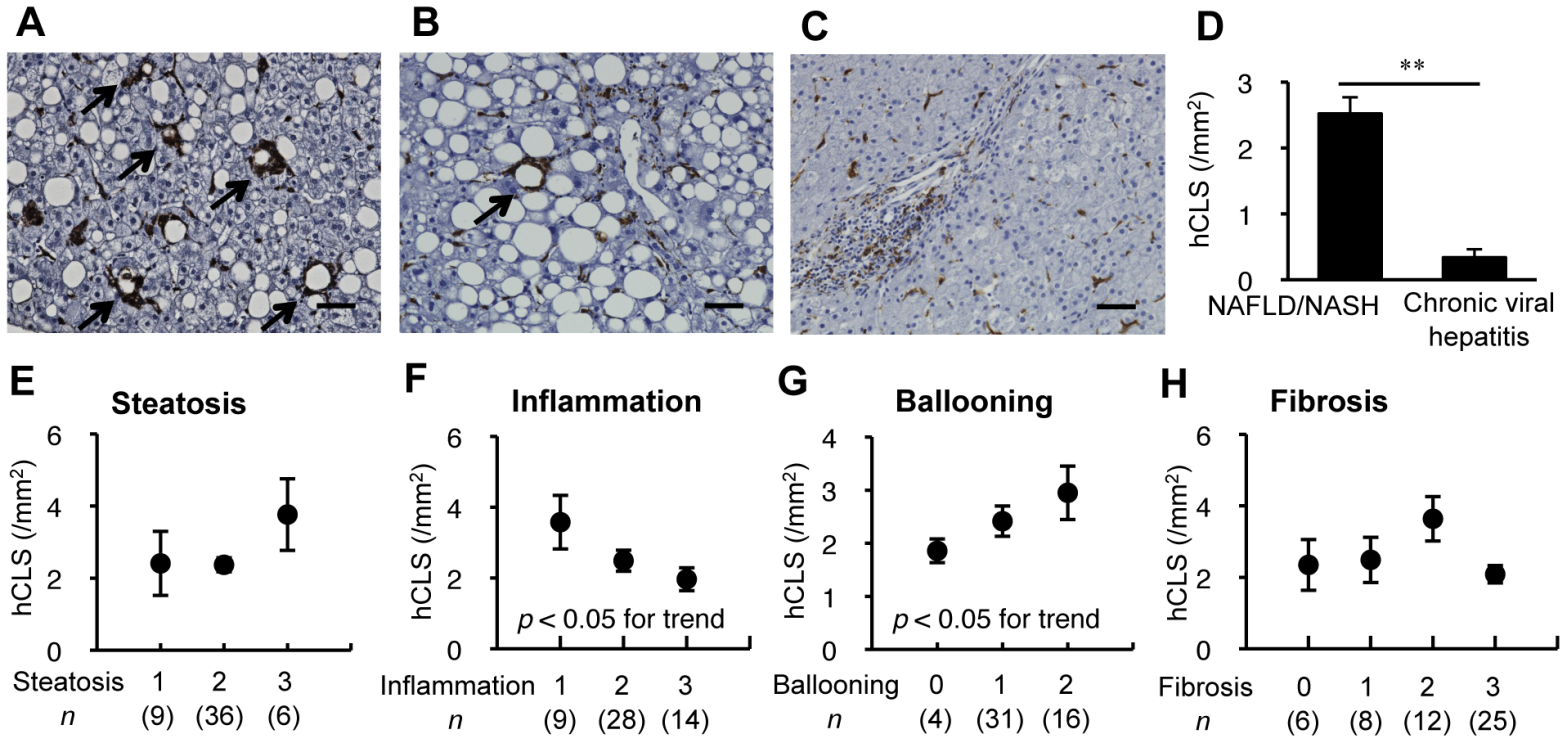
hCLS in human NASH. (A–C) Representative liver sections stained with CD68 antibody in patients with NASH (A), simple hepatic steatosis (B), and chronic viral hepatitis (C). Arrows indicate hCLS. (D) hCLS number in patients with NAFLD/NASH, and chronic viral hepatitis. Correlation of hCLS number with scores for hepatic steatosis (E), lobular inflammation (F), ballooning degenetation (G), and fibrosis stage (H) in patients with NAFLD/NASH. Scale bars, 50 µm. ** *P*<0.01.

To elucidate the clinical implications of hCLS, we next performed macrophage immunostaining using liver biopsy specimens from patients with NAFLD/NASH and chronic viral hepatitis caused by hepatitis B and C viruses. There were no significant differences in age and plasma AST and ALT concentrations between the patients. Body mass index was significantly higher in patients with NAFLD/NASH relative to those with chronic viral hepatitis (Table 2). CD68 immunostaining revealed macrophage aggregation constituting hCLS in the liver from NAFLD/NASH patients (Figure 4A, 4B, and 4D), which was rarely observed in patients with chronic viral hepatitis (Figure 4C and 4D). We further examined the correlation of the number of hCLS with the scores for hepatic steatosis, lobular inflammation, ballooning degeneration and the fibrosis stage in patients with NAFLD/NASH (Figure 4E–4H). The number of hCLS tended to be high in the patients with massive hepatic steatosis, which did not reach a statistical significance (Figure 4E) and there was a negative trend between the number of hCLS and the score for lobular inflammation (Figure 4F). Interestingly, the number of hCLS was positively associated with the score for ballooning degeneration, which is a hallmark for hepatocyte injury (Figure 4G). The patients with fibrosis stage 2 showed the highest number of hCLS (Figure 4H), which is consistent with our observations that most of the MC4R-KO mice fed a WD for 20 weeks exhibited liver fibrosis corresponding to fibrosis stage 2 in the scoring system for human NASH. These observations indicate that human NAFLD/NASH also exhibits hCLS formation, which may be associated with hepatocyte injury.

**Table 2:** Clinical data of patients with NAFLD/NASH and chronic viral hepatitis.

	NAFLD/NASH	Chronic viral hepatitis
Age	51.2±2.0	56.7±2.8
Sex (male/female)	18/33	9/6
BMI	29.1±0.7	23.3±0.9^**^
AST (IU/L)	78.7±6.9	53.3±9.4
ALT (IU/L)	122.7±11.5	82.5±20.3

± SE. ^**^ *P*<0.01. NAFLD, non-alcoholic fatty liver disease; NASH, non-alcoholic steatohepatitis; BMI, body mass index; AST, aspartate aminotransferase; ALT, alanine aminotransferase. Data are expressed as the mean

## Discussion

NASH is a severe form of NAFLD, and can progress to cirrhosis and hepatocellular carcinoma. However, it is currently unclear how simple hepatic steatosis progresses to NASH. Since macrophages play a variety of roles during the process of inflammation such as production of proinflammatory cytokines and chemokines, recruitment of immune cells, phagocytosis of dead cells, and production and degradation of extracellular matrices, macrophages should be involved in the pathogenesis of NASH. In this study, using a novel mouse model of NASH that reflects a liver condition of human NASH, we observed that macrophages aggregate to constitute hCLS surrounding hepatocytes with large lipid droplets. We have also demonstrated for the first time that the number of hCLS is positively correlated with the extent of liver fibrosis. In this regard, it is noteworthy that hCLS is clearly observed in the liver of patients with NAFLD/NASH. hCLS was also observed in other experimental models of steatohepatitis induced by a long term-HFD feeding or methionine choline-deficient diet. These observations suggest that hCLS would be a common histological feature for steatohepatitis.

Since hepatic macrophages exacerbate steatosis and lipid-mediated injuries in the liver [38], [39], it is important to know how macrophages contribute to the pathogenesis of NASH. Evidence has accumulated suggesting that macrophages play an important role in liver fibrosis *in vivo*. For instance, Kupffer cell inactivation or macrophage depletion results in lower scarring and reduced activation of hepatic stellate cells in the carbon tetrachloride-induced liver fibrosis [10], [33], [40]. In contrast, macrophage depletion at the onset of fibrosis resolution retards extracellular matrix degradation [10]. Infusion of autologous bone marrow cells including macrophages are clinically effective to repair and regenerate liver cirrhosis [41], [42]. All the reports point to the functionally distinct subpopulations of macrophages in the liver during the progression and recovery of liver fibrosis. Studies with clodronate liposomes suggest that macrophages in hCLS express inflammatory and fibrogenic genes at higher levels than those scattered in the liver from MC4R-KO mice fed a WD. Histologically, hCLS formation precedes the development of collagen deposition and is located close to fibrogenic lesions. Moreover, the number of hCLS is positively correlated with the extent of liver fibrosis. It is, therefore, conceivable that hCLS promotes liver fibrosis, which may be involved in the progression from simple steatosis to NASH.

In this study, we show that hCLS-constituting macrophages are positive for CD11c and may engulf dead hepatocytes and residual lipids in their phagosomes. The histological features are reminiscent of CLS in obese adipose tissue, in which CD11c-positive M1 macrophages surround and scavenge dead adipocytes and residual lipids [25], [27]. It is also known that ablation of CD11c-positive cells leads to a marked decrease in adipose tissue inflammation and normalizes insulin sensitivity without affecting body weight [43]. However, little is known about the involvement of CD11c-positive macrophages in fibrogenesis. Recent evidence has pointed to the role of intimate crosstalk between parenchymal and interstitial cells in the pathogenesis of chronic inflammatory diseases [14], [44]. It is now recognized that endogenous stress signals, which are referred to as “danger signals” released from necrotic cells and damaged tissues, are sensed by the innate immune system, thereby inducing sterile inflammation [14], [44]. Thus, the interaction between dying hepatocytes and hCLS-constituting macrophages may be key to understand the molecular mechanisms underlying the development of liver fibrosis in NASH. Interestingly, treatment with clodronate liposomes failed to deplete macrophages in hCLS in MC4R-KO mice on a WD, suggesting the impaired phagocytic function. This is consistent with the super-paramagnetic iron oxide magnetic resonance imaging study showing defective phagocytic function in macrophages in the liver of NASH patients [45]. Further studies are required to understand how macrophages in hCLS affect hepatic stellate cells or fibroblasts to promote fibrosis in the liver.

In this study, we demonstrate that hCLS is observed in patients with NAFLD/NASH. Interestingly, there was a positive association between the number of hCLS and the score for ballooning degeneration, which is consistent with our histological data in MC4R-KO mice that macrophages constituting hCLS may scavenge the residual lipid droplets of dead hepatocytes. On the other hand, the number of hCLS was not positively associated with the scores for hepatic steatosis and lobular inflammation. These findings support the notion that hCLS is related to the local inflammation around dying hepatocytes. In this regard, recent evidence has also pointed to the existence of hCLS in human NASH. Rensen *et al.* showed that myeloperoxidase-positive Kupffer cells and neutrophils surround steatotic hepatocytes to constitute hCLS in human NASH [46]. Ioannou *et al.* also showed that CD68-positive macrophages form hCLS around lipid droplets containing cholesterol crystals in human NASH, which was not observed in patients with simple hepatic steatosis [47]. As the degrees of steatosis were not equivalent between NAFLD/NASH and HCV patients in this study, further studies with the full spectrum of patients from simple steatosis to NASH, and/or HCV patients with steatosis are required to elucidate whether hCLS is specific for NAFLD/NASH. Given that hCLS formation precedes the development of collagen deposition in MC4R-KO mice, hCLS could be a prognostic marker for NAFLD/NASH. Since our clinical study is the cross-sectional evaluation of hCLS in patients with NAFLD/NASH, it is interesting to perform a prospective follow-up study to investigate the possibility that hCLS predicts disease progression from simple steatosis to NASH.

Clusters of macrophages have been reported as microgranulomas in human NAFLD/NASH, and those with lipid droplets are referred to as lipogranulomas [48], [49]. Lipogranulomas are characterized by a lipid droplet surrounded by macrophages and occasionally eosinophils, lymphocytes, and neutrophils [46], [48], [50], which may share morphological characteristics with hCLS in this study. To the best of our knowledge, this is the first report to elucidate the potential role of hCLS in liver fibrosis in NASH. In our mouse model, the number of microgranulomas was quite low (0.18±0.03/mm^2^) relative to that of hCLS (25.61±0.18/mm^2^) (M. Itoh *et al.* unpublished observations). It is, therefore, technically difficult to examine the correlation of microgranulomas with the histological scores. On the other hand, the number of microgranuloma in the human biopsies was 3.67±0.35/mm^2^, which was much larger than that in MC4R-KO mice. This might be due to the difference in species or the degree of steatosis. In this regard, we do not exclude the possibility that microgranulomas are involved in liver fibrosis in human NASH. In line with this, there are several reports showing the increased number of microgranulomas in NASH relative to simple steatosis and the correlation between the number of microgranulomas and the extent of liver fibrosis [51], [52]. Accordingly, it is interesting to know the difference of the role of hCLS and microgranulomas in liver fibrosis.

This study demonstrates for the first time that hCLS is a unique histological feature correlated with liver fibrosis in our mouse model of NASH. We also observed increased number of hCLS in the liver of NAFLD/NASH patients. Our data suggest that in the development of NASH, macrophages constitute hCLS, where they interact with dead hepatocytes and fibrogenic cells, thereby accelerating inflammation and fibrosis in the liver. Collectively, our data provide evidence that hCLS is involved in the development of hepatic inflammation and fibrosis, thereby suggesting its pathophysiologic role in disease progression from simple steatosis to NASH.

## Supporting Information

Figure S1 **hCLS formation in mouse model of steatohepatitis.** Sirius red (A) and F4/80 (B) stainings in the liver of MC4R-KO mice fed a high-fat diet (HFD) for 20 weeks. (C) Correlation of fibrosis area with hCLS number. Sirius red (D) and F4/80 (E) stainings in the liver from wildtype mice fed a HFD for one year. Sirius red (F) and F4/80 (G) stainings in the liver from wildtype mice fed a methionine and choline-deficient diet for 4 weeks. hCLS was indicated by arrows. Scale bars, 50 µm.
(PDF)
