# Supplementary figures and images for: Hepatic Crown-Like Structure: A Unique Histological Feature in Non-Alcoholic Steatohepatitis in Mice and Humans
Source: PLoS One. 2013 Dec 11;8(12):e82163. doi: 10.1371/journal.pone.0082163 (PMC3859576; doi:10.1371/journal.pone.0082163)

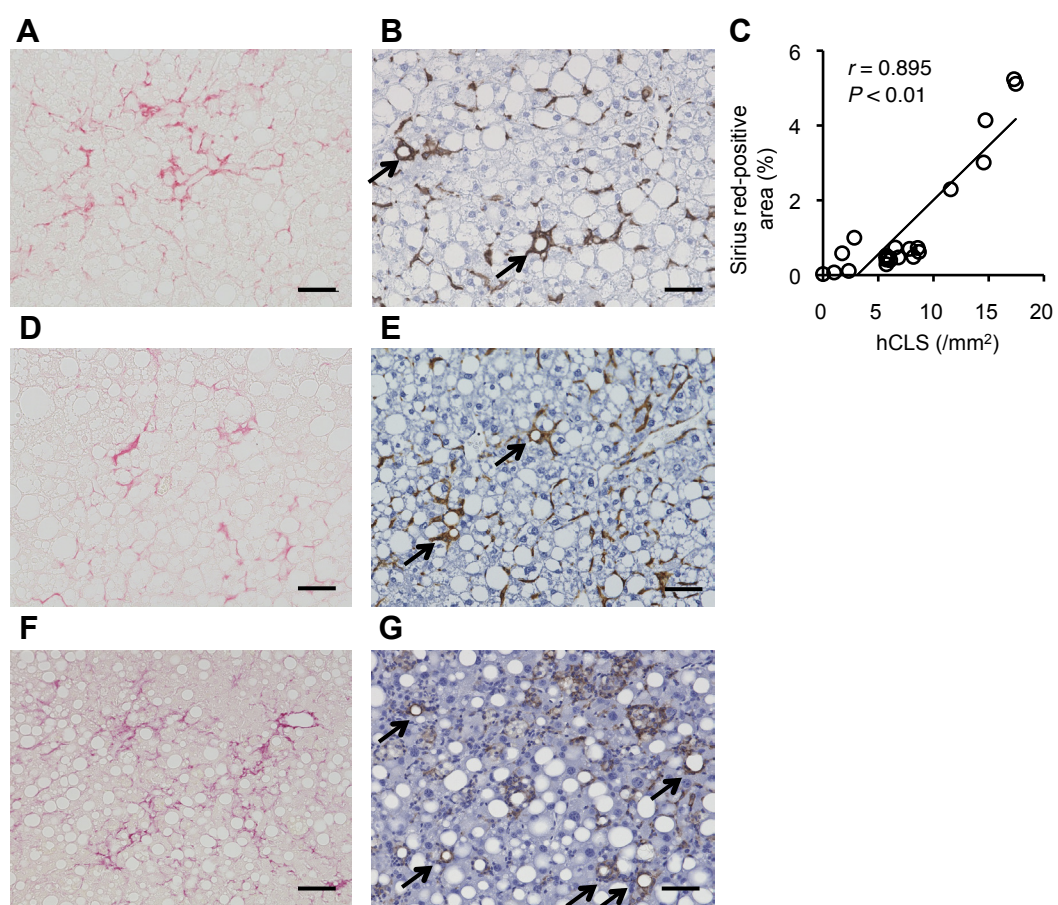

Figure S1

Supplement: Figure S1 — hCLS formation in mouse model of steatohepatitis. Sirius red (A) and F4/80 (B) stainings in the liver of MC4R-KO mice fed a high-fat diet (HFD) for 20 weeks. (C) Correlation of fibrosis area with hCLS number. Sirius red (D) and F4/80 (E) stainings in the liver from wildtype mice fed a HFD for one year. Sirius red (F) and F4/80 (G) stainings in the liver from wildtype mice fed a methionine and choline-deficient diet for 4 weeks. hCLS was indicated by arrows. Scale bars, 50 µm. (PDF) [file pone.0082163.s001.pdf]
